# Supplementary material for: Towards complete assignment of the infrared spectrum of the protonated water cluster H+(H2O)21
Source: Nat Commun. 2021 Oct 22;12:6141. doi: 10.1038/s41467-021-26284-x (PMC8536673; doi:10.1038/s41467-021-26284-x)
Supplement: Supplementary file 1 — Supplementary Information [file 41467_2021_26284_MOESM1_ESM.pdf]

SUPPLEMENTARY MATERIAL

Towards Complete Assignment of the Infrared Spectrum of the Protonated Water Cluster $H^+(H_2O)_{21}$

J. Liu, et al.

Supplementary Methods

Relation of VQDPT2 with Other Vibrational Methods

Here, we briefly describe how VQDPT2 is related to other vibrational methods in the literature, namely, the second-order vibrational Møller-Plesset (VMP2) (also knowns as the correlation corrected VSCF (cc-VSCF))[1, 2], the second-order vibrational perturbation theory (VPT2)[3], and the vibrational configuration interaction (VCI)[4].

When the *P* space is reduced to one configuration, i.e., the target configuration (**n**), Eq. (7) of the main text is rewritten as,

$$\left(H_{eff}^{(2)}\right)_{nn} = E_{\mathbf{n}}^{\mathrm{VMP2}} = \sum_{\mathbf{q}\neq\mathbf{n}} \frac{\left|\langle\Phi_{\mathbf{n}}^{\mathrm{VSCF}}|\hat{H}_v|\Phi_{\mathbf{q}}^{\mathrm{VSCF}}\rangle\right|^2}{E_{\mathbf{n}}^{(0)} - E_{\mathbf{q}}^{(0)}} \tag{1}$$

This equation is equivalent to VMP2. Therefore, VQDPT2 coincides with VMP2 if the target state has no quasi-degenerate states. Further simplification of Eq. (1) gives VPT2 by replacing the VSCF solution with the harmonic solution, and by approximating the PES to a quartic force field (QFF),

$$E_{\mathbf{n}}^{\mathrm{VPT2}} = \sum_{\mathbf{q}\neq\mathbf{n}} \frac{\left|\langle\Phi_{\mathbf{n}}^{\mathrm{HO}}|\delta V|\Phi_{\mathbf{q}}^{\mathrm{HO}}\rangle\right|^2}{E_{\mathbf{n}}^{\mathrm{HO}} - E_{\mathbf{q}}^{\mathrm{HO}}} \tag{2}$$

where $\Phi_{\mathbf{n}}^{\mathrm{HO}}$ is the harmonic oscillator wavefunction and

$$E_{\mathbf{n}}^{\mathrm{HO}} = \sum_{i=1}^{f} \left(n_i + \frac{1}{2}\right)\omega_i \tag{3}$$

$$\delta V = \sum_{ijk} c_{ijk} Q_i Q_j Q_k + \sum_{ijk} c_{iijk} Q_i^2 Q_j Q_k \tag{4}$$

The integrals in Eq. (2) can be carried out analytically, so that VPT2 is cost effective compared to other anharmonic vibrational methods. VPT2 is available in Gaussian16,[5] and has been widely used. However, the application of VPT2 to protonated water clusters is severely limited, because (1) the PES is highly anharmonic and beyond the applicability of QFF, and (2) the vibrational resonance caused by the motion of proton makes the perturbative expansion unstable [the energy difference in the denominator of Eq. (2) is close to zero]. The drawbacks of VPT2 have been pointed out in the previous report.[6]

The VCI wavefunction is represented by a linear combination of VSCF configuration functions,

$$\Psi_{\mathbf{n}}^{\mathrm{VCI}} = \sum_{\mathbf{m}} c_{\mathbf{nm}} \Phi_{\mathbf{m}}^{\mathrm{VSCF}} \quad (5)$$

The expansion coefficients are obtained by solving a secular equation,

$$\mathbf{Hc} = \mathbf{Ec} \quad (6)$$

$$\mathbf{H}_{\mathbf{mm'}} = \left\langle \Phi_{\mathbf{m}}^{\mathrm{VSCF}} \middle| \hat{H}_v \middle| \Phi_{\mathbf{m'}}^{\mathrm{VSCF}} \right\rangle \quad (7)$$

VCI converges to the exact solution if all possible VSCF configurations are used in Eq. (5). In practice, the expansion is truncated at some level to meet the computational cost. For example, the VCI space was constructed by restricting the level of excitation to 11, 10, 9, and 8 for one-, two-, three-, and four-mode excitations, respectively, in VCI calculations of a $H_9O_4^+$ moiety in $H^+(H_2O)_{21}$.[6, 7] The VCI dimension was about 140,000.

The first-order VQDPT in Eq. (6) of the main text is equivalent to VCI truncated to *the P space*, and VQDPT2 is an improvement over the truncated VCI. In the original article,[8, 9] the benchmark calculations have shown that VQDPT2 calculations with N_{gen} =3 and $\lambda_{\max}$ = 4 were comparable to large VCI calculations. In the present work, the number of *P* and *Q* space configurations was about 900 and 300 million, respectively, in the VQDPT2 calculations of $H^+(H_2O)_{21}$.

Vibrational calculations based on local coordinates

Although conventional vibrational calculations have been carried out in terms of normal coordinates, local coordinates have gained more attention in recent years. We have developed optimized coordinate VSCF (oc-VSCF) method[10], which yields a set of variationally optimal coordinates to describe the system. Application to $H_9O_4^+$ has shown that coordinates localized to each molecule (H_3O^+ and H_2O) is better than delocalized, normal coordinates.[11] The result is consistent with Wang and Bowman,[12] who proposed to employ coordinates localized to each molecule for VCI calculations of molecular clusters.

Very recently, we have developed VQDPT2 calculations based on local coordinates.[13] The coordinates are obtained by diagonalizing the Hessian matrix in a block of user-specified group of atoms. The PES is represented as a sum of the intra-group anharmonic PES (V_g) and inter-group harmonic coupling ($c_{gg'}$) as,

$$V \simeq \sum_g V_g(\boldsymbol{Q}_g) + \sum_{g>g'} c_{gg'} \boldsymbol{Q}_g \boldsymbol{Q}_{g'} \quad (8)$$

where g is an index of the groups. The intra-group PES is generated in a conventional way, whereas the inter-group coupling is truncated at the harmonic level. Because the inter-group coupling is obtained from the Hessian matrix, the cost of PES generation is drastically reduced. The validity of the approximation was tested through an application to a strong hydrogen bond network in biomolecules.[13]

Supplementary Note 1

Assignments of O-H stretches of the neutral water molecules of structure a1

The relatively isolated peak at 3595 cm^{-1} with a shoulder around 3575 cm^{-1} in the experimental spectrum (denoted as **a**, see Fig. 2) is well reproduced by the VQDPT2/CCD calculation which predicts the corresponding peak and shoulder at ca. 3550 and 3529 cm^{-1} (see Fig. 3), respectively. These features stem from the O-H stretching modes of DDA^d-type water molecules. The doublet of the feature **a** is caused by the different orientations of DDA^d-type water molecules relative to $AADD^i$, as shown in Figs. 3B and 3C. The experimental peak around 3525 cm^{-1} (denoted as **b**) corresponding to the calculated features ca. 3472 and 3494 cm^{-1} is predominately attributed to $AADD^s$-type water molecules (Figs. 3D and 3E). A plateau around 3450 cm^{-1} and a prominent band near 3390 cm^{-1} in the experimental spectrum (denoted as **c** and **d**, respectively) are mixtures of different types of water molecules. Hydrogen-bonded O-H groups of AAD-type water molecules (Fig. 3F) make the largest contribution to the feature **c**, whereas the symmetric O-H stretching modes of $AADD^s$-type and DDA^h-type water molecules (Figs. 3G and 3H, respectively) have the second largest contribution. The asymmetric O-H stretching mode of DDA^h-type water molecules and hydrogen-bonded OH groups of AAD-type water molecules (Figs. 3I and 3J, respectively) contribute mainly to the feature **d**. The peaks calculated at 3361, 3323, and 3284 cm^{-1} (denoted as **e** and **g**, respectively) are due to hydrogen-bonded OH groups of AAD-type water molecules (Fig. 3K). The feature around 3304 cm^{-1} (denoted as **f**) is assigned to the asymmetric O-H stretching mode of the interior $AADD^i$ water molecule (Fig. 3L). The remaining bands (denoted as **h** and **i**) are attributed to the overtones and combination tones of water bending modes.

Supplementary Note 2

Comparison of O-H stretches of the neutral water molecules in structures a2 – a4.

For those unique features appeared in the O-H stretching region of neutral water molecules in the structures a2, a3 and a4 in Fig. 5C, we can also give clear assignments. For the structure a2, the peak at 3461 cm^{-1} stems from the resonance of the symmetric and asymmetric O-H stretching vibrations of the $AADD^s$-type water. The relatively weak feature at 3360 cm^{-1} is a combination band of the bending vibrations of H_3O^+ and $AADD^s$-type water. For the structure a3, both of the 3432 and 3470 cm^{-1} features are results of the resonance of the symmetric and asymmetric O-H stretching vibrations of the $AADD^s$-type water. The peak at 3421 cm^{-1} arises from the resonance of the asymmetric O-H stretching vibrations of the $AADD^s$-type water with the bonded O-H vibration of AAD-type water. The peaks around 3390 cm^{-1} are mixtures of combination bands including the frustrated rotation of H_3O^+, the rocking and bending vibrations of the DDA^h-type water molecules. For the structure a4, the peak at 3466 cm^{-1} is also attributed to the resonance of the symmetric and asymmetric O-H stretching vibrations of the $AADD^s$-type water molecule. The 3458 cm^{-1} feature is due to the resonance of the symmetric and asymmetric O-H stretching vibrations of the DDA^h-type water molecule. The feature at 3450 cm^{-1} manifests the bonded O-H vibrations of AAD-type water. The peak at 3368 cm^{-1} is a combination of the bending vibrations of H_3O^+ and DDA^h-type water. The weak 3341 cm^{-1} feature is attributed to the symmetric O-H stretching vibration of the $AADD^i$-type water.

Supplementary Note 3

CCSD calculation for 1MR-PES

The correction of higher level electronic correlation effects by using CCSD/aug-cc-pVTZ, though only for 1MR-PES, would further refine the quality of the PES.[11] The anharmonic spectrum computed with the multiresolution PES is very close to the CCD/aug-cc-pVDZ result, as shown in Supplementary Fig. 3, in which only the relative intensities show slight difference for the bands around 1800 and 2400 cm^{-1}. This result substantiates the effectiveness of the CCD/aug-cc-pVDZ level in computing and interpreting the IR spectral features for quantitative band assignment. Therefore, the structural implications drawn in this work provide convincing and definitive proof for the assignment of the band features associated with the excess proton and its coupling with the neutral water network.

Supplementary Note 4

Cartesian coordinates (Å) of the optimized structure a1

O	4.6568501729	4.8312518895	-0.5115888210
H	4.1619663335	4.7979228377	-1.4063961309
H	3.9714995271	5.0337662672	0.2203724575
H	5.0395036382	3.8981413837	-0.3329374515
O	2.9722046740	5.2106409770	1.3733853262
H	2.8523246887	4.3384907916	1.7943939367
H	2.0670009158	5.5005425574	1.1611877465
O	0.7749054537	4.2388930681	-1.7087763714
H	1.0984538146	3.4127830833	-1.2957142351
H	0.0465753100	3.9443684845	-2.2818131971
O	0.1970259262	1.6589035465	3.2673882367
H	0.0722722717	1.8621629898	4.2020341238
H	1.0701485965	2.0350815549	3.0282459535
O	3.9577932764	1.0195342727	-1.9041009981
H	3.9344932228	0.0481946822	-1.9548788536
H	3.9328986425	1.3509305644	-2.8193610390
O	2.4956284134	2.5651477995	2.1093469885
H	2.2733283433	2.3224499376	1.1875350300
H	3.3237975272	2.0833351451	2.2849409572
O	3.3918940722	2.3885822720	-4.2434709166
H	3.7181175455	2.3777061774	-5.1508655267
H	2.4688242912	2.0599310659	-4.2865634203
O	0.9010308573	1.3298900340	-4.0782099691
H	0.0847965946	1.7847500366	-3.8190921299
H	0.8069541719	0.4152397958	-3.7712268076
O	3.2042336248	-0.9994144630	1.1454751892
H	2.2937517949	-1.0594614553	1.4719923506
H	3.1959105034	-1.3369739282	0.2367515611
O	3.3289110883	4.7492680571	-2.6932086633
H	2.3806603515	4.7183693891	-2.4657990909
H	3.4571543331	3.9980846969	-3.2998133375
O	-2.3188357472	1.8186895155	-0.6530964746
H	-3.2531627573	1.6501028480	-0.4887124584
H	-2.0243216730	2.4004350795	0.0857261525
O	0.4294096480	-0.8772412089	2.0801078680
H	0.3051784132	-0.0812049154	2.6327847487
H	0.0666751908	-1.6079146552	2.5932622703
O	5.5554259337	2.4673389437	-0.1782859355
H	5.4376227467	2.0043748937	0.6700070786
H	5.1217246109	1.8902066408	-0.8344018848
O	-1.3193350759	2.8665495278	-3.0555177361
H	-1.8059223639	2.5099011271	-2.2883927749

H	-1.9863605319	3.2563596862	-3.6315436088
O	1.7757206859	1.9694865735	-0.4913536030
H	2.5263084465	1.6067741672	-1.0039841728
H	1.0957312878	1.2685262636	-0.5108885819
O	-1.3200386064	3.2745853378	1.3836222488
H	-0.8877563358	2.7904315347	2.1045499177
H	-0.8234486732	4.1022042608	1.2787871526
O	3.2928079361	-1.6886223821	-1.6741493272
H	3.7650844943	-2.4802344208	-1.9565010199
H	2.4340099024	-1.7109987728	-2.1347599409
O	0.7313564130	-1.2646688634	-2.7646813748
H	0.1569466052	-1.9520646204	-3.1219359426
H	0.2719640710	-0.9282423877	-1.9698060696
O	0.2628834695	5.5862096008	0.6473236487
H	-0.1160148432	6.4714878897	0.5998171350
H	0.3472784132	5.2778107581	-0.2768530917
O	4.8148212123	0.9708318820	2.0878080261
H	5.4257039561	0.6565189177	2.7642987744
H	4.3344191381	0.1777209489	1.7686431841
O	-0.1960018307	0.0003536385	-0.5051402728
H	-1.0464984551	0.4761595177	-0.5199527631
H	-0.1365264239	-0.4183301589	0.3723786378

Cartesian coordinates (Å) of the optimized structure a2

O	-1.2547240307	-0.3363670120	-4.1823543937
H	-0.5903986961	-1.0838537148	-4.3972042043
H	-1.8446352277	-0.6593456553	-3.4113040806
H	-0.7088330116	0.4576267210	-3.8342841991
O	0.2137514610	1.5483043029	-3.2979251379
H	-0.0424520193	2.0171447441	-2.4836730619
H	1.0922530853	1.1690885792	-3.1027465905
O	-1.2483937325	0.0592042934	-0.0681649729
H	-0.4499085708	-0.4492794374	-0.3185810537
H	-1.4331594218	-0.2132134306	0.8474260842
O	2.0908692369	1.6926446105	0.7396888585
H	1.3201166865	2.1072923204	0.3213261922
H	1.7531141613	1.2044793689	1.5051163515
O	0.9552631710	-1.4004578206	-0.9152131769
H	1.5197489216	-1.6347853585	-0.1526051242
H	1.5296092235	-0.8576894233	-1.4916126699
O	2.5081467565	0.1840098073	-2.6038937262
H	2.8886445708	-0.3330549342	-3.3362911101
H	3.2504498457	0.6115707747	-2.1412738689
O	-2.3364961052	-3.7381010180	-1.2922733714
H	-1.4962335511	-3.9401942923	-1.7498807245
H	-2.9608121554	-4.4255362834	-1.5507476623

O	-1.1388117686	-3.4574298863	1.3529903179
H	-1.6091946449	-3.6279231635	0.5218974735
H	-0.3364103146	-3.9999643185	1.3309785938
O	3.9326019328	-3.1253439021	-2.2041123398
H	4.3527202133	-2.6483327478	-1.4731024003
H	3.5106231076	-3.9079277330	-1.8180976573
O	-2.6606200733	-1.0729081922	-2.1811917010
H	-2.6760705714	-2.0189515033	-1.9499026987
H	-2.3113758672	-0.6303812889	-1.3840266883
O	1.3000895834	-0.1106392653	2.8940477612
H	0.3631659641	-0.3886985688	2.8974772899
H	1.5011327743	0.1207023637	3.8083271890
O	4.3071265112	1.1613995973	-0.7081963797
H	3.5603777460	1.4217094875	-0.1248243155
H	4.8812626021	1.9343433805	-0.7566902428
O	0.3896329476	-2.2394422054	-4.6153267432
H	0.3267430724	-2.8923257127	-3.8924542897
H	1.3414614356	-2.0482196334	-4.6932126273
O	-1.3116106656	-1.0399585551	2.5318842968
H	-2.0447762137	-1.0103005114	3.1569582293
H	-1.2888809539	-1.9622850432	2.1917954439
O	0.2358788685	-3.7141238134	-2.2592045929
H	0.5143133216	-2.9262619319	-1.7488982118
H	0.9005827077	-4.3899378816	-2.0429368467
O	2.5278137545	-2.0623806748	1.2884406255
H	3.4417765651	-1.8573262360	1.0214608640
H	2.2707906528	-1.4019143174	1.9569668440
O	2.3263049777	-5.3160509290	-1.2120232505
H	2.5482436039	-6.2346174596	-1.4028421185
H	2.0991748955	-5.2821841802	-0.2641450227
O	1.5327560816	-4.6395514652	1.3944433671
H	1.9729911502	-3.7711551993	1.4881784209
H	1.7577171352	-5.1303899036	2.1939238105
O	-0.2904006527	2.5472876452	-0.7122565395
H	-0.8240358059	1.7883717360	-0.4034291266
H	-0.8253872663	3.3359807900	-0.5666151333
O	3.1666329804	-1.7830992365	-4.4375262060
H	3.5070402445	-2.3652299568	-3.7256653271
H	3.7653402086	-1.8875467475	-5.1852272150
O	4.9439018060	-1.4668761118	-0.0042318302
H	5.8459243148	-1.5889359580	0.3141886818
H	4.8801508854	-0.5235879830	-0.2513610485

Cartesian coordinates (Å) of the optimized structure a3

O	1.4232078210	2.3225513385	-2.5532777355
H	0.9211822165	1.4337201697	-2.4936088615

H	1.0545732813	2.9360521336	-1.8200644767
H	2.4033782520	2.1372744111	-2.3222044681
O	0.6244321569	3.8196688008	-0.6546804661
H	1.2937542527	3.7646930090	0.0542957515
H	-0.2240083494	3.6044537785	-0.2269769013
O	-0.8333556961	0.3335867837	0.2950719698
H	0.0781923254	0.4196970660	0.6433224869
H	-1.2084746728	-0.4233486851	0.7762033614
O	2.0873672691	3.6718687009	3.9592293363
H	1.1974671782	3.2836071478	3.8130508206
H	1.9404148635	4.4888626980	4.4490657936
O	3.4921669041	-0.6675361967	-0.6578650492
H	4.1734677938	-1.1573868888	-0.1656902690
H	3.0148486577	-1.3226600075	-1.1964688113
O	2.5759809671	3.3078495972	1.2309970793
H	3.4871486055	3.4571753626	0.9230945306
H	2.5300679172	3.6477070145	2.1416315292
O	1.5649295427	-2.2604469952	-1.8931795372
H	1.2413912522	-2.5575745751	-1.0177888134
H	1.5157726735	-3.0232684756	-2.4795943757
O	0.9613981605	-2.5818279784	0.7237033993
H	0.1159431132	-2.5236327836	1.1931195376
H	1.6078656058	-2.8947372896	1.3740105967
O	5.1611040051	1.0000673724	1.9347158419
H	4.8135836891	1.1359948154	2.8282129461
H	5.2694999294	0.0446397226	1.8187847780
O	0.1353996575	0.1330710661	-2.3038009071
H	0.6278295276	-0.7068348808	-2.3138113842
H	-0.3316922341	0.1374374237	-1.4461624224
O	-0.6301019256	-0.2951654913	4.2105537075
H	-0.8698844444	-0.4383688299	5.1334870032
H	0.3407594845	-0.4203726877	4.1654985861
O	3.8141862074	1.5370367231	4.4732467843
H	3.2813661088	2.3539541271	4.4138269253
H	4.3064897787	1.6105697278	5.2988338997
O	3.8242148669	1.7543847952	-1.9281781485
H	4.3244666800	2.3163930546	-1.3102579828
H	3.8364782578	0.8631743451	-1.5294821839
O	-1.4939426424	-1.8185733135	2.0142514466
H	-2.3227503991	-2.3022086341	2.1015352957
H	-1.3438889779	-1.3775448866	2.8713100007
O	1.7796906315	0.6243605381	1.1321902059
H	2.0954387191	1.5481397341	1.1592292298
H	2.3747976501	0.1641715093	0.5095217436
O	-0.1910846896	2.4212397291	3.2455677150
H	-0.5343950199	1.5805945958	3.5813244586

H	-0.7799771146	2.6886676138	2.5236809176
O	5.1745011263	-1.8129018938	1.2643005401
H	5.9708860471	-2.3470340873	1.1686966070
H	4.5571754912	-2.3400987596	1.8045918166
O	3.0375849259	-2.9106926017	2.7195022830
H	2.7242048427	-2.1148289787	3.1972560740
H	3.0669195471	-3.6175715418	3.3743240606
O	-1.6110191368	2.9150320400	0.7828051805
H	-2.5084228653	3.2058692745	0.5855663768
H	-1.5610672741	1.9710495514	0.5316376098
O	5.1384950834	3.0578843965	0.1818991893
H	5.9533965868	3.5641713910	0.0941758214
H	5.3347599205	2.3157741554	0.7907994341
O	2.0557762363	-0.4902253496	3.6066394815
H	2.6630167221	0.1224313973	4.0583653791
H	1.9602856318	-0.0924701933	2.7129971710

Cartesian coordinates (Å) of the optimized structure a4

O	-1.2042086313	-0.3413265243	-4.0841770401
H	-0.5464543124	-1.0895883129	-4.3155790905
H	-1.8037083235	-0.6653824560	-3.3195695707
H	-0.6603067364	0.4576620066	-3.7429074552
O	0.2447129528	1.5717031645	-3.2359968836
H	-0.0244604916	2.0316004420	-2.4209950429
H	1.1251946366	1.1957024516	-3.0441596087
O	-1.2533012743	0.0613565418	-0.0130843087
H	-0.4556730093	-0.4358336477	-0.2859449118
H	-1.4166173210	-0.2223424413	0.9033129393
O	2.0470235076	1.5815618259	0.5847785167
H	1.2894208946	2.0732930656	0.2318890787
H	1.7284722096	1.1408258594	1.3861391055
O	0.9391132699	-1.3835306588	-0.9222962774
H	1.5051097739	-1.6186286681	-0.1613276263
H	1.5201304637	-0.8467191458	-1.4969856442
O	2.5344194260	0.1691514849	-2.6183545571
H	2.8983443996	-0.3560574843	-3.3529343896
H	3.2924989389	0.5689905357	-2.1577828274
O	-2.3384879600	-3.7879725093	-1.3781202292
H	-1.4772722903	-3.9552773139	-1.8096973765
H	-2.9257582928	-4.4960051351	-1.6651102872
O	-1.1214050557	-3.3798124008	1.2199473088
H	-1.6220748182	-3.5955062597	0.4181628754
H	-0.3316575729	-3.9407970251	1.2115085780
O	3.8324302373	-3.1000077915	-2.1588025413
H	4.2858362568	-2.6427420487	-1.4356538309
H	3.4633896441	-3.9139190139	-1.7852142838

O	-2.6525913913	-1.0830376865	-2.1227102901
H	-2.6793102631	-2.0358976944	-1.9229352723
H	-2.3087187838	-0.6617022984	-1.3118988026
O	1.2982245936	-0.0711051344	2.8459416791
H	0.3709054349	-0.3756016974	2.8853191524
H	1.5167597848	0.2002384394	3.7449590859
O	4.3554238326	1.1500275896	-0.7357725132
H	3.5826561701	1.4165124487	-0.1921721858
H	4.9199350261	1.9291299425	-0.7893380319
O	0.4086614086	-2.2520650345	-4.5854320885
H	0.3601759171	-2.9034042598	-3.8600963204
H	1.3590068352	-2.0715392983	-4.6929088637
O	-1.3009726849	-1.0560603222	2.5666654956
H	-2.0258439941	-1.0525345044	3.2015139132
H	-1.2907650840	-1.9583607021	2.1785281986
O	0.2622492718	-3.7240704134	-2.2414516818
H	0.5244961728	-2.9262269109	-1.7387557526
H	0.9295177683	-4.3919070240	-2.0077317240
O	2.5172988129	-2.0478615656	1.2757282120
H	3.4332756931	-1.8513068981	1.0096312027
H	2.2665772221	-1.3796168271	1.9380593211
O	2.3226264552	-5.3431295248	-1.1762921680
H	2.5224986669	-6.2698323508	-1.3495958366
H	2.0847402376	-5.2892194324	-0.2321717916
O	1.4936902196	-4.5995681210	1.3866825970
H	1.9405350714	-3.7339207456	1.4762018494
H	1.6682616274	-5.0647593403	2.2133462916
O	-0.3618752956	2.5693456564	-0.6674113056
H	-0.8757598461	1.7952627134	-0.3632399537
H	-0.9152364184	3.3418076397	-0.5079429911
O	3.2028888215	-1.8068089527	-4.4578307780
H	3.5182757562	-2.3913041796	-3.7380303781
H	3.8209608293	-1.9184526916	-5.1884082156
O	4.9331688274	-1.4730559015	0.0030142410
H	5.8330989431	-1.6139926788	0.3193200550
H	4.8901425343	-0.5295227001	-0.2465940879

Cartesian coordinates (Å) of the MP2 optimized structure

O	2.22256817	-3.07659791	-0.84825649
H	2.24936305	-2.56567148	-0.01104716
H	2.59239246	-2.46167217	-1.51329254
O	0.44634426	-0.16114936	-2.68415051
H	0.28320122	-0.06319270	-1.71408374
H	0.21753874	0.71661437	-3.05082618
O	3.49370210	0.62487548	1.86423228
H	4.36128902	0.40891699	2.23275139

H	3.00857868	-0.23839314	1.77308468
O	-2.50410049	-0.97164029	0.59849474
H	-2.91540778	-0.38365315	1.26365833
H	-3.06655448	-0.92686173	-0.20579444
O	1.87966776	-1.43339715	1.36687887
H	1.15449447	-0.92644130	0.92443148
H	1.43789072	-1.87429849	2.12517121
O	-3.52948465	-0.72653611	-1.92000704
H	-4.41480566	-0.78727401	-2.30241056
H	-3.25546113	0.22272742	-2.01084806
O	-2.66325646	1.80181852	-1.88642758
H	-1.86897207	2.13327292	-2.34410288
H	-2.69377690	2.27395394	-1.03486485
O	-0.59009986	0.11576373	3.59466077
H	0.08157045	0.81257165	3.48725690
H	-1.43497432	0.49155356	3.28673105
O	-1.32206692	-2.29578214	-2.75791394
H	-0.72337482	-1.53552449	-2.91898776
H	-2.17756727	-1.87538367	-2.53794158
O	1.69158877	3.36185080	-1.38107355
H	2.21502256	4.16733715	-1.49149270
H	2.35916578	2.62674506	-1.25100626
O	1.50589934	2.07373426	3.08117099
H	2.32239050	1.61468474	2.77201467
H	1.78796778	2.65157322	3.80299581
O	-1.31880261	-3.40578917	1.14869103
H	-0.79335687	-3.22478404	1.95352786
H	-1.87348646	-2.60434042	1.04037088
O	-0.21272288	2.51980759	-3.19300405
H	0.49089732	2.94904098	-2.65515281
H	-0.23779165	3.00156114	-4.03060174
O	-0.01404159	-0.00555409	0.00999415
H	-0.92798056	-0.32150254	0.21685518
H	0.00205435	0.93575022	0.29414576
O	3.34163863	1.37640062	-0.90369077
H	3.49343891	1.10973020	0.02232508
H	3.37106077	0.55937314	-1.43919193
O	-3.09709166	0.98113129	2.48958334
H	-3.85760664	1.09023354	3.07622991
H	-3.04551400	1.80576175	1.95620439
O	-2.54171017	3.04219442	0.73789580
H	-2.85302350	3.95366313	0.82326863
H	-1.55670167	3.08368309	0.80534808
O	3.02640002	-0.93803231	-2.52376138
H	3.52775018	-1.06469006	-3.34060080
H	2.11981358	-0.65164813	-2.79301677

O	0.20038796	-2.42308754	3.31859374
H	0.32777400	-2.87054892	4.16532983
H	-0.15747426	-1.52369622	3.53100746
O	0.12599146	2.66988910	0.77543578
H	0.70284502	3.07494291	0.08984617
H	0.65291018	2.64575362	1.60379606
O	-0.19249145	-3.86748315	-1.09211841
H	-0.68118001	-3.25944314	-1.75965001
H	0.78862840	-3.56517381	-1.04399429
H	-0.61426095	-3.71683097	-0.16739398

Supplementary Table 1. The assignments of the bands associated with H_3O^+. Notations: frustrated rotation (rot), umbrella vibration (umb), H-O-H bending (bend), libration (lib), sym (symmetric O-H stretching), asym (asymmetric O-H stretching), overtone (Ovtn), combination band (Comb).

Exp./cm^{-1}	VQDPT2/cm^{-1}	Weight	Intensity	Mode
940	943	0.201	158	H_3O^+ rot
*	951	0.297	21	H_3O^+ rot
*	983	0.731	5	H_3O^+ rot
1220	1267	0.930	307	H_3O^+ umb
*	1728	0.101	4	H_3O^+ bend
*	1733	0.197	1	H_3O^+ bend
1746	1791	0.035	74	H_3O^+ asym^1
*	1801	0.102	63	Comb(H_3O^+ rot + H_2O lib)
*	1812	0.366	60	Comb(H_3O^+ rot + H_2O lib)
*	1839	0.088	53	Comb(H_3O^+ rot + H_2O lib)
*	1918	0.085	51	Comb(H_3O^+ rot + H_2O lib)
1949	1949	0.051/asym^1<br>0.032/asym^2<br>0.140/Comb	160	H_3O^+ asym^1, H_3O^+ asym^2,<br>Comb(H_3O^+ umb + H_3O^+ rot + H_2O lib)
*	1954	0.278	77	Comb(H_3O^+ umb + H_2O lib)
*	1974	0.166	57	Comb(H_3O^+ umb + H_2O lib)
*	2023	0.382	81	Comb(H_3O^+ umb + H_2O lib)
2015	2035	0.113	267	H_3O^+ asym^2
*	2052	0.054/asym^1<br>0.195/Comb	185	H_3O^+ asym^1,<br>Comb(H_3O^+ umb + H_2O lib)
*	2068	0.026/asym^1<br>0.429/Comb	95	H_3O^+ asym^1,<br>Comb(H_3O^+ umb + H_2O lib)
*	2138	0.024/asym^2<br>0.261/Comb	91	H_3O^+ asym^2,<br>Comb(H_3O^+ umb + H_2O lib)
2220	2261	0.221/asym^2<br>0.360/Comb	529	H_3O^+ asym^2,<br>Comb(H_3O^+ umb + H_3O^+ rot)
*	2286	0.172/asym^1<br>0.333/Comb	406	H_3O^+ asym^1,<br>Comb(H_3O^+ umb + H_3O^+ rot)
*	2308	0.037/asym^1<br>0.485/Comb	90	H_3O^+ asym^1,<br>Comb(H_3O^+ rot + H_2O lib)
2400	2409	0.296	194	H_3O^+ sym
*	2417	0.132/sym<br>0.302/Comb	83	H_3O^+ sym,<br>Comb(H_3O^+ bend + H_2O lib)
*	2420	0.066/sym<br>0.195/Comb	62	H_3O^+ sym,<br>Comb(H_3O^+ bend + H_2O lib)
*	2438	0.025/asym^2<br>0.432/Comb	70	H_3O^+ asym^2,<br>Comb(H_3O^+ bend + H_2O lib)
*	2513	0.739	59	Ovtn (H_3O^+ umb)
2720	2714	0.040/asym^2<br>0.612/Comb	115	H_3O^+ asym^2,<br>Comb(H_3O^+ bend + H_3O^+ rot)
*	2726	0.061/asym^1	168	H_3O^+ asym^1,

		0.586/Comb		Comb(H_3O^+ bend + H_3O^+ rot)
MAE[x]	24.0			

[x]The mean absolute error.

Supplementary Table 2. The assignments of the librational modes of neutral water.

Type	Exp./cm^{-1}	VQDPT2/cm^{-1}	Weight	Intensity
**DDA^{h}**	*	696	0.348	91
**AAD**	*	707	0.397	52
**AAD**	*	714	0.235	14
**DDA^{h}**	*	720	0.367	74
**AAD**	*	725	0.288	23
**DDA^{h}**	*	746	0.459	91
**AADD^{s}**	*	777	0.409	82
**AAD**	*	795	0.554	81
**AAD**	~840	855	0.248	293
**AAD**	*	862	0.379	63
**AADD^{s}**	*	893	0.209	30
**AAD**	*	908	0.265	190
**AAD**	*	923	0.319	39
**AAD**	*	963	0.487	49

Supplementary Table 3. The assignments of the bands (**a-i**, in Fig. 3) associated with the OH stretching motions of the neutral water molecules. Notations: overtone (Ovtn), combination band (Comb).

Type	Mode	HB partner[x]	Exp./cm^{-1}	VQDPT2/cm^{-1}	Weight	IR intensity	Band
**AAD**	Ovtn (bends)	*	3110	3166	0.352	188	**i**
**AAD**+**DDA^{d}**	Comb (bends)	*	3206	3269	0.121	29	**h**
**AAD**	OH	DDA^{d}	3230	3284	0.368	332	**g**
**AADD^{i}**	asym	AADD^{s}, AADD^{s}	3304	3304	0.526	514	**f**
**AAD**	OH	DDA^{d}	*	3323	0.860	646	**e**
**AAD**	OH	DDA^{d}	3335	3327	0.660	317	
**AAD**	OH	AADD^{s}	*	3361	0.449	545	
**DDA^{h}**	sym	AADD^{s}, AAD	*	3378	0.106	214	**d**
**AAD**	OH	AADD^{s}	*	3383	0.345	636	
**DDA^{h}**	asym	AADD^{s}, AAD	3390	3388	0.126	636	
**AAD**	OH	AADD^{s}	*	3392	0.157	226	
**AADD^{s}**	asym	AAD, AAD	*	3402	0.363	732	
**AADD^{s}**	sym	AAD, AAD	*	3411	0.145	278	
**AAD**	OH	AAD	*	3429	0.711	425	**c**
**AAD**	OH	AAD	*	3438	0.818	431	
**DDA^{h}**	sym	AADD^{s}, AAD	*	3444	0.377	407	
**AAD**	OH	AAD	3450	3450	0.568	697	
**DDA^{h}**	asym	AADD^{s}, AAD	*	3452	0.189	229	
**DDA^{h}**	sym	AADD^{s}, AAD	*	3453	0.188	281	
**AADD^{s}**	sym	AAD, AAD	*	3459	0.442	254	
**AADD^{s}**	OH	AAD	3525	3472	0.668	451	**b**
**AADD^{s}**	asym	AAD, AAD	*	3476	0.383	305	
**AADD^{s}**	OH	AAD	*	3494	0.772	277	
**DDA^{d}**	sym	AAD, AAD	*	3527	0.913	174	**a**
**DDA^{d}**	asym	AAD, AAD	3575	3529	0.998	506	
**DDA^{d}**	sym	AAD, AAD	*	3538	0.917	106	
**DDA^{d}**	sym	AAD, AAD	*	3539	0.887	221	
**DDA^{d}**	asym	AAD, AAD	*	3547	0.977	452	
**DDA^{d}**	asym	AAD, AAD	3595	3550	0.976	564	
MAE[y]				32.7			

[x]The type of hydrogen-bonded water molecules directly involved in each vibrational mode.
[y]The mean absolute error.

Supplementary Table 4. Comparison of the harmonic frequencies associated with normal and local coordinates.

Character	Normal	Local	normal - local		Character	Normal	Local	normal - local
water OH	3898.0	3897.0	1.0		H_3O^+ HOH	1826.8	1804.9	21.9
	3896.0	3895.5	0.5			1822.1	1800.5	21.6
	3893.8	3893.8	0.0		water HOH	1743.8	1727.1	16.7
	3893.5	3893.2	0.3			1737.0	1724.4	12.7
	3891.2	3890.8	0.5			1726.9	1721.8	5.1
	3887.9	3887.5	0.4			1721.0	1721.1	-0.1
	3886.4	3886.3	0.1			1719.4	1719.7	-0.3
	3885.5	3885.1	0.4			1717.8	1709.8	8.0
	3883.0	3882.7	0.3			1711.8	1698.8	13.0
	3822.0	3821.0	1.0			1706.6	1695.0	11.6
	3816.4	3815.5	0.9			1700.5	1692.8	7.6
	3803.0	3802.2	0.8			1696.9	1689.5	7.3
	3739.1	3734.5	4.6			1685.6	1683.9	1.7
	3732.9	3728.0	4.9			1685.0	1683.1	1.9
	3728.6	3727.0	1.6			1677.9	1681.0	-3.2
	3722.0	3722.2	-0.2			1676.5	1680.6	-4.1
	3717.7	3716.6	1.2			1674.5	1678.8	-4.4
	3716.7	3714.3	2.4			1671.3	1676.4	-5.1
	3710.5	3700.4	10.2			1670.2	1676.1	-5.9
	3701.5	3697.5	4.1			1667.4	1671.5	-4.1
	3698.0	3696.7	1.3			1661.8	1671.1	-9.2
	3691.3	3695.5	-4.2			1658.2	1670.8	-12.6
	3673.5	3668.7	4.8		H_3O^+ umb	1356.9	1338.1	18.9
	3665.3	3653.3	12.1		H_3O^+ rot	1018.0	939.2	78.9
	3660.1	3648.1	12.0			1000.8	901.7	99.0
	3647.2	3642.8	4.4			989.6	899.5	90.1

	3640.9	3638.3	2.6		H_2O lib	976.0	899.1	76.9
	3638.5	3636.1	2.4			949.0	865.0	84.0
	3636.4	3632.1	4.3			942.9	856.7	86.2
	3624.5	3629.5	-5.1			936.1	848.9	87.3
	3620.0	3628.6	-8.6			932.0	844.1	88.0
	3616.6	3620.0	-3.4			901.4	841.9	59.6
	3605.9	3614.8	-8.9			893.3	823.9	69.4
	3603.4	3593.6	9.8			874.9	817.3	57.5
	3585.9	3590.4	-4.5			841.3	808.2	33.0
	3568.9	3579.5	-10.6			824.8	800.2	24.6
	3554.3	3577.1	-22.7			803.3	788.4	14.9
	3552.1	3557.0	-4.9			779.5	788.2	-8.7
	3549.1	3554.1	-5.0			770.5	776.6	-6.1
	3476.1	3478.8	-2.7			713.1	758.7	-45.5
H_3O^+ OH	2832.1	2831.5	0.6			692.7	731.6	-38.9
	2715.6	2715.7	-0.1			680.7	725.5	-44.9
	2700.3	2700.2	0.1			653.2	707.9	-54.7
						634.0	656.0	-22.0
						613.8	619.3	-5.5
						599.4	614.5	-15.1

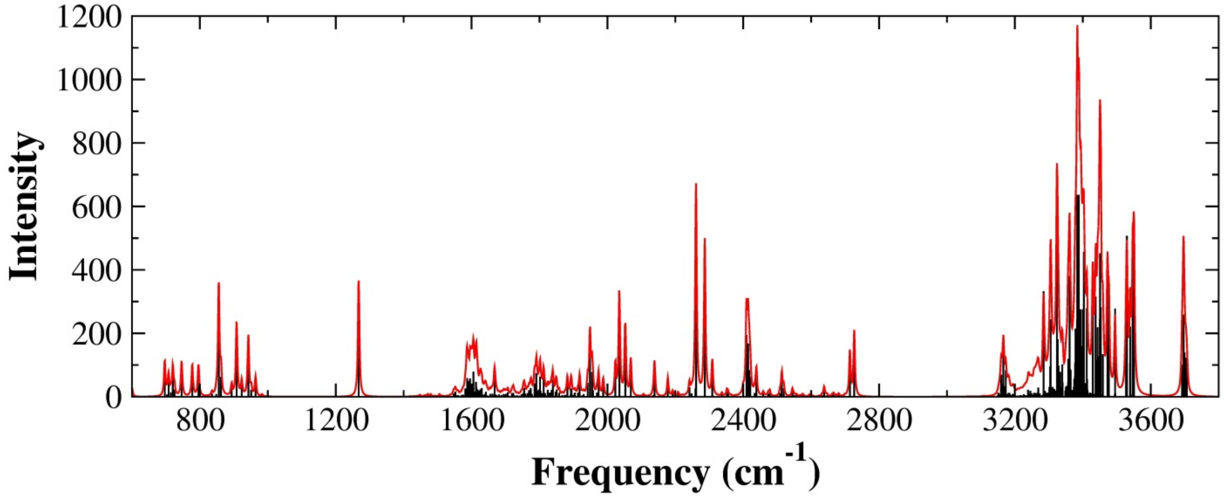

Supplementary Figure 1. IR stick (black drop lines) and Lorentz broadened (red line) spectra of the isomer 1 of the $H^{+}(H_2O)_{21}$ cluster at the Eigen state computed by VQDPT2 method at the fragment-based CCD/aug-cc-pVDZ level.

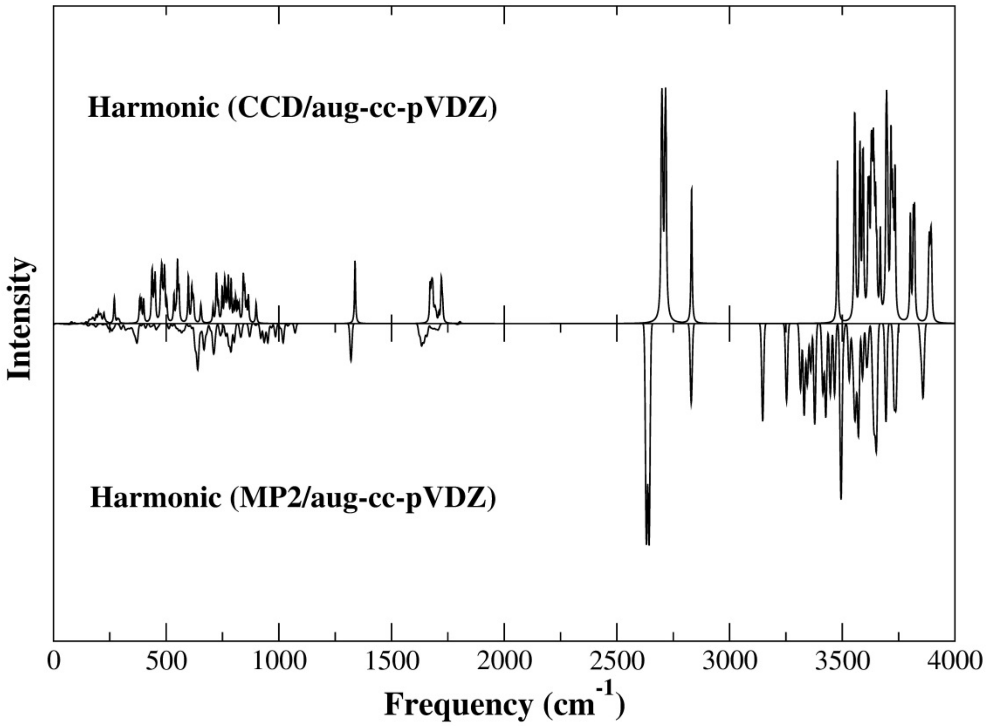

Supplementary Figure 2. Comparison of the harmonic IR spectra of the $H^+(H_2O)_{21}$ cluster in the Eigen state computed at the CCD (fragment-based calculation) and MP2 (full system calculation) levels, respectively, by using the aug-cc-pVDZ basis set. The MP2 result was based on the optimized low-lying energy isomer from ref. 14.

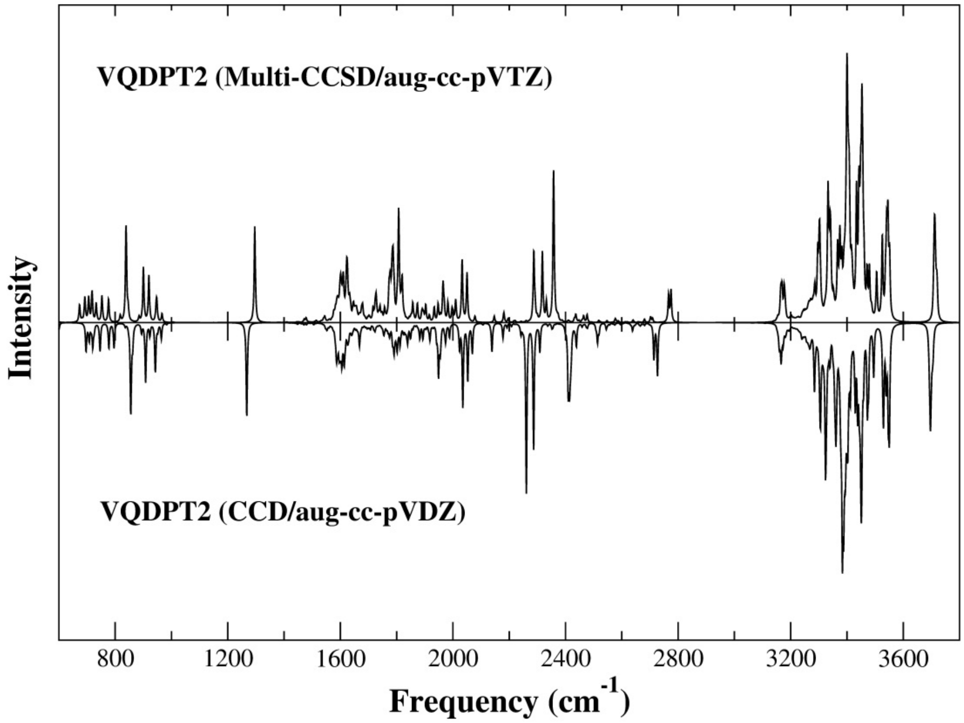

Supplementary Figure 3. Comparison of the anharmonic spectra computed through the VQDPT2 method with different PESs. "CCD/aug-cc-pVDZ" denotes all 1MR, 2MR, and 3MR grid points calculated at the fragment-based CCD/aug-cc-pVDZ level. "Multi-CCSD/aug-cc-pVTZ" represents the 1MR grid potential functions calculated at the fragment-based CCSD/aug-cc-pVTZ level, and 2MR and 3MR grid potential functions calculated at the fragment-based CCD/aug-cc-pVDZ level.

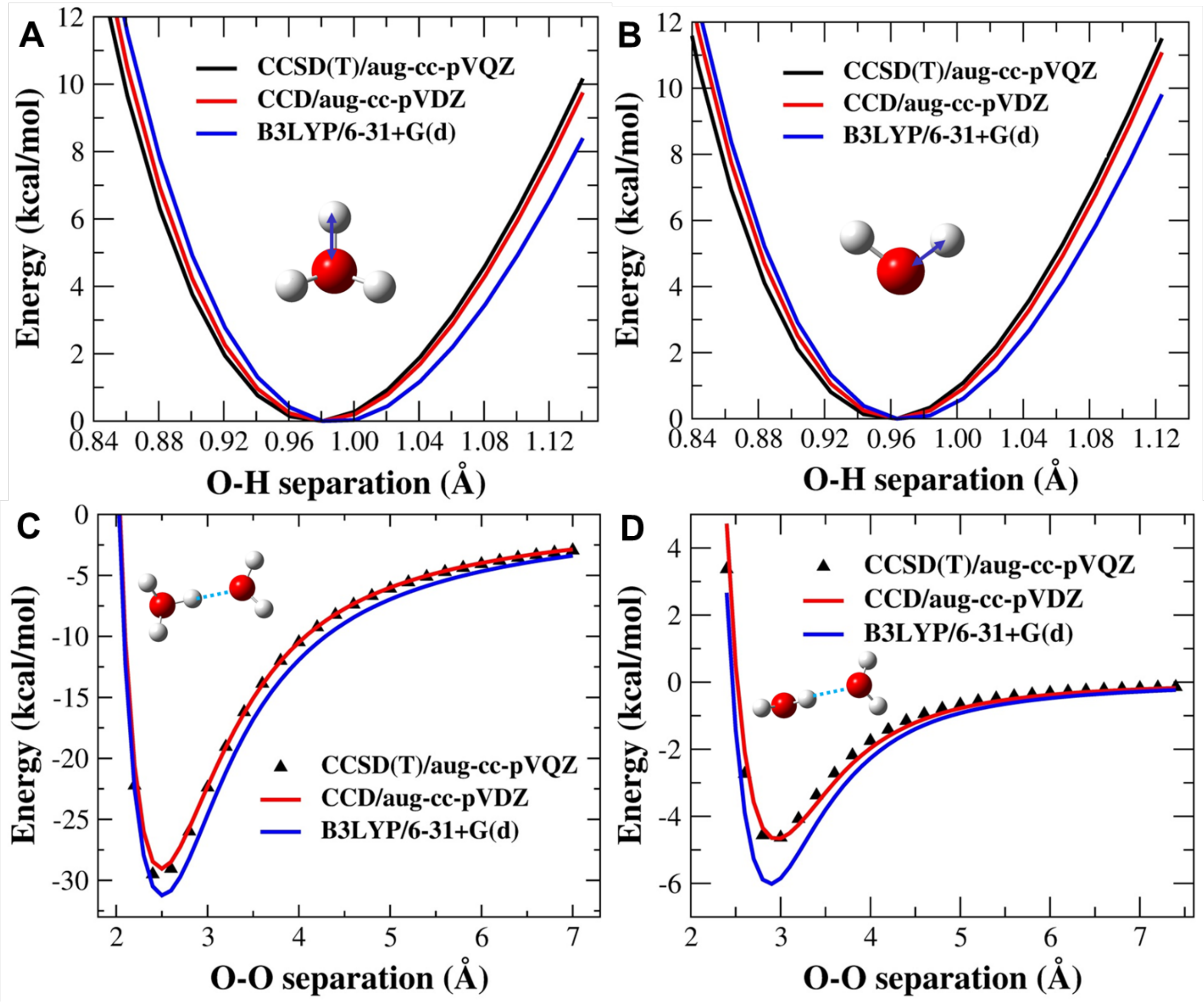

Supplementary Figure 4. The OH bond stretching potential energy surface of (A) H_3O^+ cation and (B) H_2O molecule, and the molecular interaction potential energy surface of (C) $H_3O^+\cdots H_2O$ and (D) $H_2O\cdots H_2O$ calculated at the CCD/aug-cc-pVDZ level in comparison with the results obtained from the B3LYP/6-31+G(d) level, with reference to the CCSD(T)/aug-cc-pVQZ results.

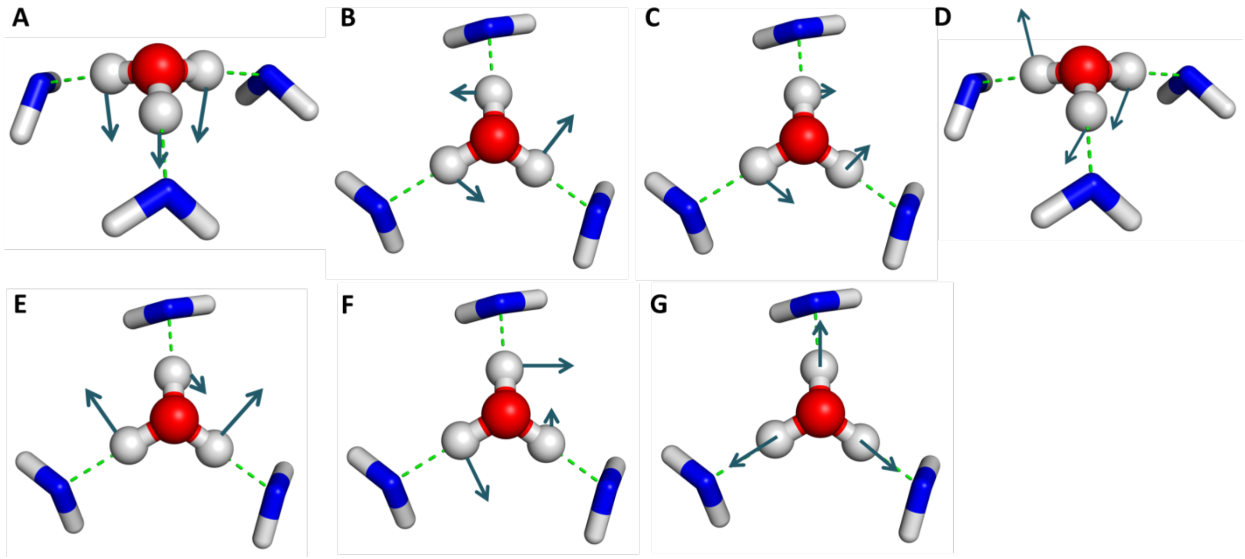

Supplementary Figure 5. The (A) umbrella vibration ($\nu^{u}_{H_3O^+}$), (B), (C), (D) frustrated rotation ($\nu^{r}_{H_3O^+}$), (E), (F) H-O-H bending ($\nu^{b}_{H_3O^+}$), and (G) symmetric O-H stretching modes of H_3O^+ ($\nu^{s}_{H_3O^+}$).

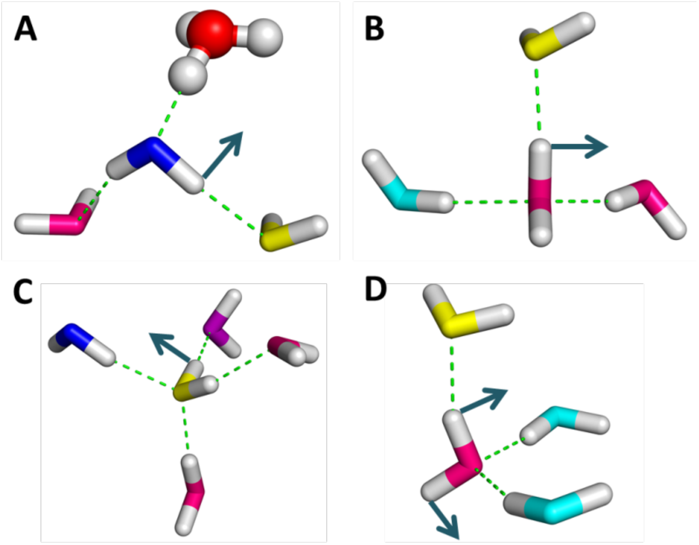

Supplementary Figure 6. The librations of (A) the DDA-type water molecule around H_3O^+ in a single hydrogen-bond acceptor configuration, (B) the AAD-type and (C) AADD-type water molecules far from H_3O^+. (D) The intramolecular bending vibrations of neutral water molecule. Color coding: H_3O^+ (red); DDA-type water molecule hydrogen-bonded with the hydronium ion (DDA^h, blue); DDA-type water molecule away from the hydronium ion (DDA^d, turquoise); AAD-type water molecule (pink); four-coordinated AADD water molecule in the interior ($AADD^i$, purple); and four-coordinated AADD water molecule at the surface ($AADD^s$, yellow).

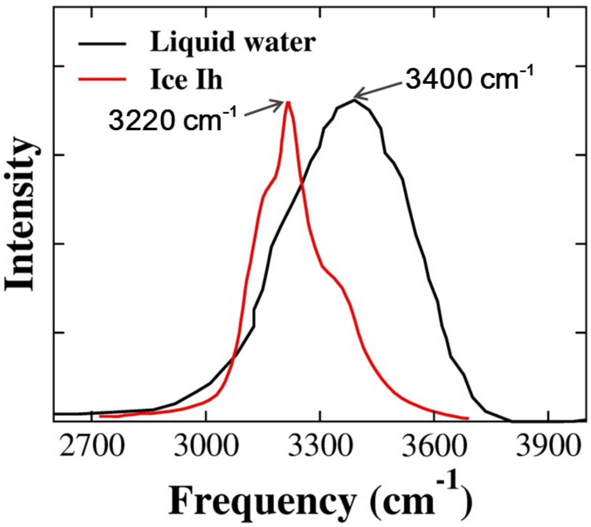

Supplementary Figure 7. Comparison of the experimental bulk IR spectra of ice Ih and liquid water in the O-H stretching region. These data are extracted from refs.15, 16.

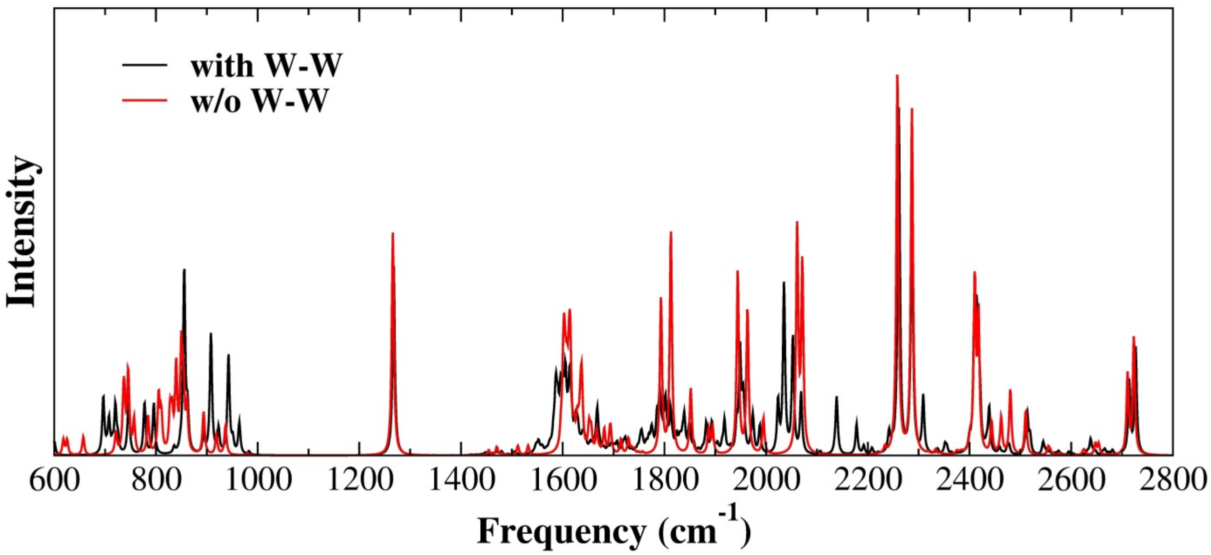

Supplementary Figure 8. Comparison of the IR spectra obtained by VQDPT2 with and without the harmonic coupling between water molecules in the PES. Note that coupling between DDA^h are included in both cases, so as the $H_3O^+(H_2O)_3$ moiety to have all inter-molecular couplings included.

Supplementary References:

1. Christiansen, O. Moller-Plesset perturbation theory for vibrational wave functions. *J. Chem. Phys.* **119**, 5773-5781 (2003).
2. Norris, L.S., Ratner, M.A., Roitberg, A.E. & Gerber, R.B. Moller-Plesset perturbation theory applied to vibrational problems. *J. Chem. Phys.* **105**, 11261-11267 (1996).
3. Barone, V. Anharmonic vibrational properties by a fully automated second-order perturbative approach. *J. Chem. Phys.* **122**, 014108 (2005).
4. Christoffel, K.M. & Bowman, J.M. Investigations of Self-Consistent Field, SCF CI and Virtual State Configuration-Interaction Vibrational Energies for a Model 3-mode System. *Chem. Phys. Lett.* **85**, 220−224 (1982).
5. Frisch, M.J. et al. Gaussian 16, Revision A.03, Gaussian, Inc., Wallingford CT. (2016).
6. Wang, H. & Agmon, N. Reinvestigation of the Infrared Spectrum of the Gas-Phase Protonated Water Tetramer. *J. Phys. Chem. A* **121**, 3056-3070 (2017).
7. Yu, Q. & Bowman, J.M. Tracking Hydronium/Water Stretches in Magic $H_3O^+(H_2O)_{20}$ Clusters through High-level Quantum VSCF/VCI Calculations. *J. Phys. Chem. A* **124**, 1167-1175 (2020).
8. Yagi, K., Hirata, S. & Hirao, K. Vibrational quasi-degenerate perturbation theory: applications to fermi resonance in CO_2, H_2CO, and C_6H_6. *Phys. Chem. Chem. Phys.* **10**, 1781-1788 (2008).
9. Yagi, K. & Otaki, H. Vibrational quasi-degenerate perturbation theory with optimized coordinates: Applications to ethylene and trans-1,3-butadiene. *J. Chem. Phys.* **140**, 084113 (2014).
10. Yagi, K., Keceli, M. & Hirata, S. Optimized coordinates for anharmonic vibrational structure theories. *J. Chem. Phys.* **137**, 204118 (2012).
11. Yagi, K. & Thomsen, B. Infrared Spectra of Protonated Water Clusters, $H^+(H_2O)_4$, in Eigen and Zundel Forms Studied by Vibrational Quasi-Degenerate Perturbation Theory. *J. Phys. Chem. A* **121**, 2386-2398 (2017).
12. Wang, Y.M. & Bowman, J.M. Ab initio potential and dipole moment surfaces for water. II. Local-monomer calculations of the infrared spectra of water clusters. *J. Chem. Phys.* **134**, 154510 (2011).

13. Yagi, K. & Sugita, Y. Anharmonic Vibrational Calculations Based on Group-Localized Coordinates: Applications to Internal Water Molecules in Bacteriorhodopsin. *J. Chem. Theory Comput.* **17**, 5007-5020 (2021).
14. Xantheas, S.S. Low-lying energy isomers and global minima of aqueous nanoclusters: Structures and spectroscopic features of the pentagonal dodecahedron $(H_2O)_{20}$ and $(H_3O)^+(H_2O)_{20}$. *Can. J. Chem. Eng.* **90**, 843-851 (2012).
15. Bertie, J.E. & Whalley, E. Infrared spectra of ices Ih and Ic in the range 4000 to 350 cm^{-1}. *J. Chem. Phys.* **40**, 1637 (1964).
16. Bertie, J.E. & Lan, Z.D. Infrared intensities of liquids XX: The intensity of the OH stretching band of liquid water revisited, and the best current values of the optical constants of $H_2O(l)$ at 25°C between 15,000 and 1 cm^{-1}. *Appl. Spectrosc.* **50**, 1047-1057 (1996).
